# Anti‐inflammatory effect of lanoconazole on 12‐*O*‐tetradecanoylphorbol‐13‐acetate‐ and 2,4,6‐trinitrophenyl chloride–induced skin inflammation in mice

**DOI:** 10.1111/myc.13034

**Published:** 2019-11-27

**Authors:** Aki Nakamura, Hideya Uratsuji, Yoshihito Yamada, Kei Hashimoto, Naoki Nozawa, Tatsumi Matsumoto

**Affiliations:** ^1^ Drug Development Research Laboratories Maruho Co., Ltd. Kyoto Japan

**Keywords:** antifungal agents, anti‐inflammatory agents, chemokines, CXC, dermatitis:contact, dermatitis:irritant, dermatomycoses, lanoconazole, pharmacology

## Abstract

**Background:**

Lanoconazole (LCZ) is a topical antifungal agent clinically used to treat fungal infections such as tinea pedis. LCZ has not only antifungal effects but also anti‐inflammatory effects, which have the potential to provide additional clinical benefits. However, the characteristic features of the inhibitory effects of LCZ on skin inflammation remain unclear.

**Objective:**

We evaluated the inhibitory effects of topical application of LCZ, and compared the effects of LCZ with those of other antifungal agents including liranaftate, terbinafine and amorolfine.

**Methods:**

Each antifungal agent was topically applied on 12‐*O*‐tetradecanoylphorbol‐13‐acetate‐induced irritant dermatitis and 2,4,6‐trinitrophenyl chloride‐induced contact dermatitis in mice (BALB/c). The ear thickness, myeloperoxidase activity and inflammatory mediator contents were evaluated.

**Results:**

LCZ dose‐dependently suppressed 12‐*O*‐tetradecanoylphorbol‐13‐acetate‐induced irritant dermatitis, suppressed the production of neutrophil chemotactic factors such as keratinocyte‐derived chemokine and macrophage inflammatory protein‐2, and inhibited neutrophil infiltration to the inflammation site. Moreover, 1% LCZ reduced the ear swelling in mice with 2,4,6‐trinitrophenyl chloride‐induced contact dermatitis in accordance with the inhibition of interferon‐γ production. The inhibitory potency of LCZ on these types of dermatitis in mice was stronger than that of other types of antifungal agents.

**Conclusion:**

The anti‐inflammatory effects of LCZ were exerted through the inhibition of inflammatory mediator production. These effects may contribute to the relief of dermatitis symptoms in patients with tinea pedis.

## 1 INTRODUCTION

Tinea pedis, one of the most common skin diseases worldwide, is a superficial dermatomycosis that occurs secondary to infection by dermatophytes.^1^, ^2^, ^3^ An inflammatory response induced by the dermatophyte is often observed at the infection site in patients with tinea pedis. This response is thought to be an irritant dermatitis caused by inflammatory mediators produced by epidermal keratinocytes and leucocytes that reacted to proteases and fungal components such as β‐glucan.^4^, ^5^, ^6^, ^7^ Indeed, histological examination of the skin of patients with dermatophyte infection reveals infiltration of neutrophils and lymphocytes at the infection site.^8^

12‐*O*‐Tetradecanoylphorbol‐13‐acetate (TPA) stimulates the activation of a wide variety of intracellular pathways through activation of protein kinase C, mitogen‐activated protein kinases (MAPKs) and nuclear factor‐κB (NF‐κB) followed by the generation of inflammatory mediators including tumour necrosis factor‐α, interleukin (IL)‐1β, keratinocyte‐derived chemokine (KC), macrophage inflammatory protein (MIP)‐2 and prostaglandins.^9^ Moreover, TPA has been shown to induce a variety of histological changes in mouse (BALB/c) skin, including neutrophil infiltration in both the epidermis and dermis and epidermal hyperplasia.^10^, ^11^ In patients with tinea pedis, β‐glucan (a component of the dermatophyte cell wall) reportedly binds to the host cell surface receptor dectin‐1 and subsequently evokes the production of inflammatory mediators through activation of protein kinase C, MAPK and NF‐κB inside the host cells.^12^ Infiltration of inflammatory cells, mainly neutrophils, is also observed in the histological examination of skin affected by tinea pedis. These observations suggest that there are similarities in the intracellular mechanism between the inflammatory reaction in tinea pedis and TPA‐induced inflammation.

Lanoconazole (LCZ) is an imidazole antifungal agent having potent in vitro antifungal activity against clinical dermatophyte isolates.^13^, ^14^ The clinical use of 1% LCZ cream, solution and ointment in Japan has demonstrated its therapeutic usefulness in the treatment of various dermatomycoses including tinea pedis, tinea corporis and cutaneous candidiasis.^15^ Because tinea pedis is accompanied by inflammation at the infection site, an antifungal agent containing an anti‐inflammatory effect would be therapeutically valuable. Indeed, some antifungal agents reportedly have anti‐inflammatory effects.^16^, ^17^, ^18^ Although our previous study demonstrated that LCZ suppressed TPA‐induced irritant dermatitis in mice,^19^ the characteristic features of these anti‐inflammatory effects remain to be elucidated. We also found that LCZ inhibited the production of IL‐8, a neutrophil chemotactic factor, from epidermal keratinocytes.^19^ Therefore, to characterise the anti‐inflammatory effect of LCZ, we investigated whether LCZ affects the production of the neutrophil chemotactic factor and neutrophilic infiltration to the skin in TPA‐induced irritant dermatitis in mice and compared its inhibitory potency with that of other antifungal agents. Furthermore, elevated levels of interferon (IFN)‐γ mRNA expression and IFN‐γ‐positive cells in tinea pedis lesions suggest that a T helper cell type 1 (Th1)‐driven allergic contact dermatitis is provoked in tinea pedis.^6^, ^7^ Therefore, we also compared the inhibitory potency of LCZ on Th1‐type contact hypersensitivity induced by 2,4,6‐trinitrophenyl chloride (PC) with that of other antifungal agents in mice.

## 2 MATERIALS AND METHODS

### 2.1 Animals

Female BALB/c mice aged 7 weeks were purchased from Charles River Laboratories Japan, Inc. All of the animal experimental procedures were approved by the Ethics Committee for Animal Experiments of Maruho Co., Ltd. and conducted in accordance with the Guiding Principles for the Care and Use of Laboratory Animals at Maruho Co., Ltd.

### 2.2 Reagents

LCZ was kindly provided by Nichino Service Co., Ltd. Liranaftate (LNF) was purchased from Santa Cruz Biotechnology, Inc. Terbinafine hydrochloride (TBF) and amorolfine hydrochloride (AMO) were purchased from Sigma‐Aldrich. Other reagents used were as follows: TPA (Sigma‐Aldrich); PC (Tokyo Chemical Industry Co., Ltd); mouse CXCL1/KC Quantikine^®^ enzyme‐linked immunosorbent assay (ELISA) kit, mouse CXCL2/MIP‐2 Quantikine^®^ ELISA kit and mouse IFN‐γ Quantikine^®^ ELISA kit (R&D Systems Inc); and Fluoro MPO™ Fluorescent Myeloperoxidase Detection Kit (Cell Technology).

### 2.3 TPA‐induced irritant dermatitis

TPA‐induced irritant dermatitis was induced in mice according to a previously described method.^19^ Briefly, 10 µL of 0.01% TPA (20 µL for ear) was applied to the inner and outer surfaces of the right ear of each mouse. Negative control mice received only acetone instead of TPA. Immediately after the TPA application, 20 µL of 0.3%, 1% or 3% LCZ; 1% LNF; 1% TBF; 1% AMO; or vehicle (acetone) was applied to the same ear. The ear thickness was measured 6 hours after the application of TPA using a dial thickness gauge (Series 547‐401 customised; Mitutoyo Corporation). Thereafter, 5‐mm‐diameter punch biopsies were obtained from the skin of the right ear to measure the myeloperoxidase (MPO) activity and inflammatory mediator contents, and 3‐mm‐diameter punch biopsies were obtained from the skin of the right ear, fixed with 10% neutral formalin and embedded in paraffin. Tissue sections were stained with haematoxylin and eosin for microscopic examination.

### 2.4 PC‐induced contact hypersensitivity

The mice were sensitised and challenged with PC according to a previously described method.^19^ Sensitisation was performed by application of 100 µL of 3% PC to their shaved abdomen. Negative control mice received acetone instead of PC. Six days later, the skin of the mice was challenged to elicit a contact hypersensitivity response by the application of 10 µL of 1% PC to the inner and outer surfaces of the right ear. Next, 20 µL of 1% LCZ, 1% LNF, 1% TBF, 1% AMO or vehicle (acetone) was applied to the same ear. The ear thickness was measured 24 hours after the PC application using a dial thickness gauge (Mitutoyo Corporation). Punch biopsies of 5‐mm diameter were then obtained from the skin of the right ear to determine the IFN‐γ content.

### 2.5 Measurement of MPO activity and chemokine content in the skin

The ear biopsy was homogenised in lysis buffer (phosphate‐buffered saline containing 0.1% Tween‐20 and protease inhibitor cocktail) and centrifuged for 10 minutes (10 000 *g*, 4ºC). The MPO activity and the KC, MIP‐2 and IFN‐γ contents in the supernatants were measured according to the manufacturer's instructions.

### 2.6 Statistical analysis

Data are expressed as mean ± standard error of the mean. A significant difference between two groups was evaluated by Student's *t* test or the Aspin‐Welch *t* test following the *F*‐test. Dunnett's multiple‐comparison test was used to analyse differences among three or more groups. A *P* value of <.05 was considered statistically significant.

## 3 RESULTS

### 3.1 Anti‐inflammatory effect of LCZ on TPA‐induced irritant dermatitis in mice

**Figure 1:**
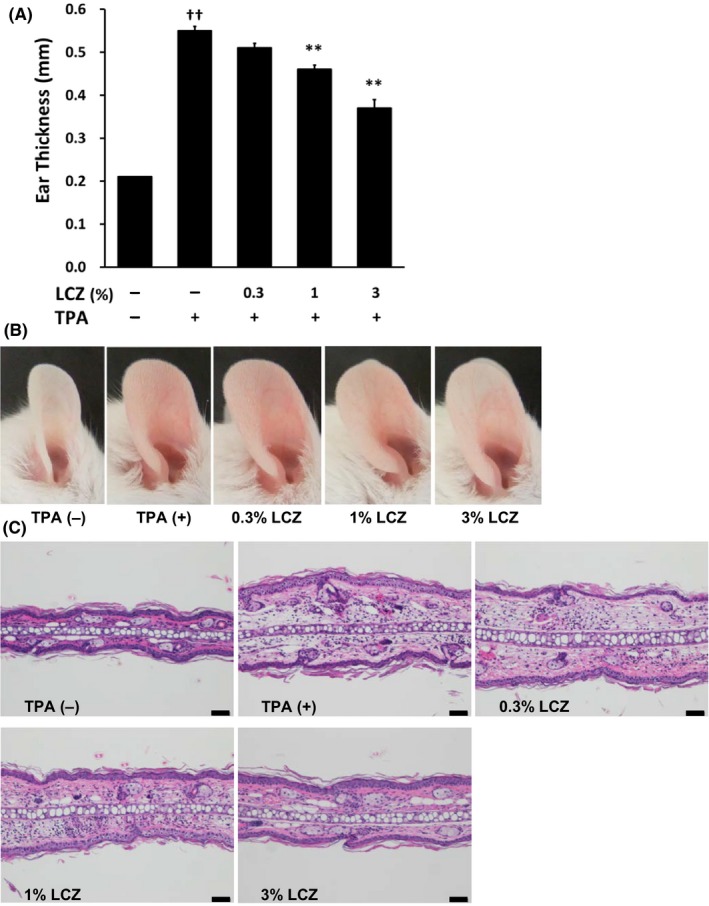
Inhibitory effect of LCZ on ear swelling induced by topical application of TPA in mice. TPA (0.01%, 20 µL) was topically applied to both sides of the right ear. LCZ (0.3%, 1% or 3%) or vehicle (acetone) was topically applied (20 µL/ear) immediately after the treatment with TPA. Negative control mice received acetone instead of TPA. (A) The ear thickness was measured 6 h after the treatment with TPA. Each column represents the mean ± standard error of the mean (n = 6). **P < .01 compared with TPA alone (Dunnett's multiple‐comparison test, two‐sided); ^††^ *P* < .01 compared with TPA (−) (Aspin–Welch *t* test, two‐sided). (B,C) Representative appearance and histological photographs of the ear 6 h after topical application of TPA in mice. Bars = 50 µm. LCZ: lanoconazole and TPA: 12‐*O*‐tetradecanoylphorbol‐13‐acetate

We first assessed the anti‐inflammatory effect of LCZ on the TPA‐induced irritant dermatitis in mice. As shown in Figure 1, topical application of TPA to the mouse ear resulted in an increase in the ear thickness. LCZ dose‐dependently reduced the TPA‐induced increase in ear thickness, and the inhibitory effect of LCZ at concentrations of 1% and 3% was significantly stronger than that of vehicle (Figure 1A,B). Additionally, histological examination showed TPA‐induced oedema and infiltration of inflammatory cells such as neutrophils and lymphocytes in the dermis with suppression of these phenomena in the LCZ treatment group (Figure 1C).

**Figure 2:**
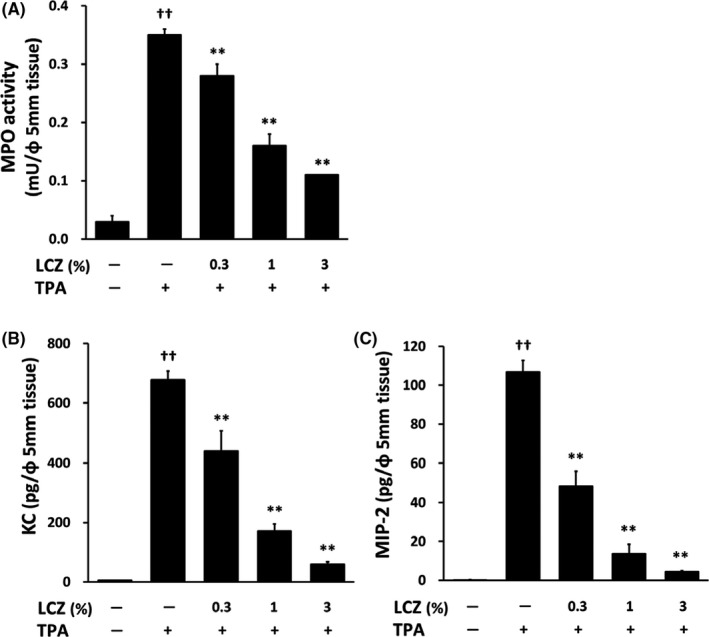
Inhibitory effect of LCZ on TPA‐induced increase in KC content, MIP‐2 content and MPO activity in the ears of mice. A 5‐mm‐diameter punch biopsy was obtained from the right ear 6 h after the treatment with acetone or TPA. (A) MPO activity in the ear biopsy was measured by fluorescence. (B, C) KC and MIP‐2 contents in the ear biopsies were measured by enzyme‐linked immunosorbent assay. Each column represents the mean ± standard error of the mean (n = 6). **P < .01 compared with TPA alone (Dunnett's multiple‐comparison test, two‐sided); ^##^ *P* < .01 compared with TPA (−) (Student's *t* test or Aspin‐Welch *t* test, two‐sided). LCZ: lanoconazole, TPA: 12‐*O*‐tetradecanoylphorbol‐13‐acetate, KC: keratinocyte‐derived chemokine, MIP‐2: macrophage inflammatory protein‐2 and MPO: myeloperoxidase

Next, we assessed MPO activity in the TPA‐applied ear as the index of neutrophil infiltration. We found that topical application of TPA induced an increase in MPO activity in the ear skin and that LCZ dose‐dependently suppressed the increase in MPO activity (Figure 2A). Furthermore, we investigated the changes in the KC and MIP‐2 contents in the TPA‐induced irritant dermatitis because mouse KC and MIP‐2 are reportedly the functional analogues of human IL‐8 (a neutrophil chemotactic factor).^20^, ^21^, ^22^ As shown in Figure 2B and 2C, the TPA application resulted in an increase in the KC and MIP‐2 protein levels in the ear skin and LCZ significantly reduced both the KC and MIP‐2 levels in a dose‐dependent manner.

**Figure 3:**
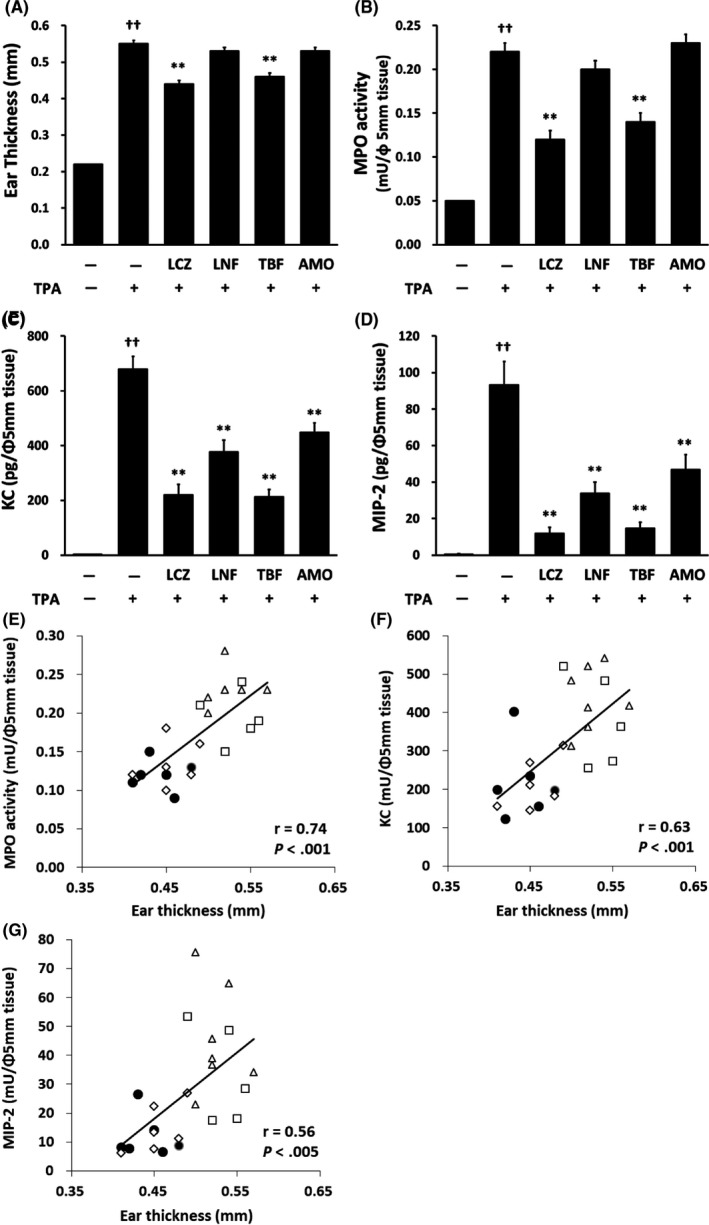
Effect of antifungal agents on ear swelling of TPA‐induced irritant dermatitis in mice. Each antifungal agent (1%) or vehicle (acetone) was topically administered (20 µL/ear) immediately after TPA application. (A) The ear thickness was measured 6 h after the application of acetone or TPA. (B–D) MPO activity, KC content and MIP‐2 content in the 5‐mm‐diameter punch biopsy obtained 6 h after TPA application were measured by fluorescence and enzyme‐linked immunosorbent assay, respectively. Each column represents the mean ± standard error of the mean (n = 6). **P < .01 compared with TPA alone (Aspin‐Welch *t* test or Student's *t* test, two‐sided); ^††^ *P* < .01 compared with TPA (−) (Aspin‐Welch* t* test, two‐sided). (E–G) The relationships between the ear thickness and MPO activity, KC content and MIP‐2 content were analysed. Closed circle: LCZ, open square: LNF, open diamond: TBF and open triangle: AMO. AMO, amorolfine; KC, keratinocyte‐derived chemokine; LCZ, lanoconazole; LNF, liranaftate; MIP‐2, macrophage inflammatory protein‐2; MPO, myeloperoxidase; TBF, terbinafine; TPA, 12‐*O*‐tetradecanoylphorbol‐13‐acetate

We then compared the anti‐inflammatory effect of LCZ with that of various antifungal agents in the TPA‐induced irritant dermatitis model. As shown in Figure 3A, treatment with LCZ or TBF significantly inhibited the increase in ear thickness induced by TPA. However, treatment with LNF or AMO showed no inhibition of the increase in ear thickness. The increase in MPO activity induced by TPA was also suppressed by treatment with LCZ or TBF, and treatment with LNF or AMO showed no inhibition of them (Figure 3B). Furthermore, the increase in the KC and MIP‐2 contents in TPA‐treated ear tissue was significantly inhibited by all antifungal agents tested, and the inhibitory effects of LCZ and TBF were stronger than those of the other agents (Figure 3C,D). We then analysed the relationship between ear thickness and each anti‐inflammatory index (MPO activity, KC content and MIP‐2 content). A significantly positive correlation with each of these anti‐inflammatory indexes was found (*r* = .74, .63 and .56, respectively).

### 3.2 Anti‐inflammatory effect of LCZ on PC‐induced contact dermatitis in mice

**Figure 4:**
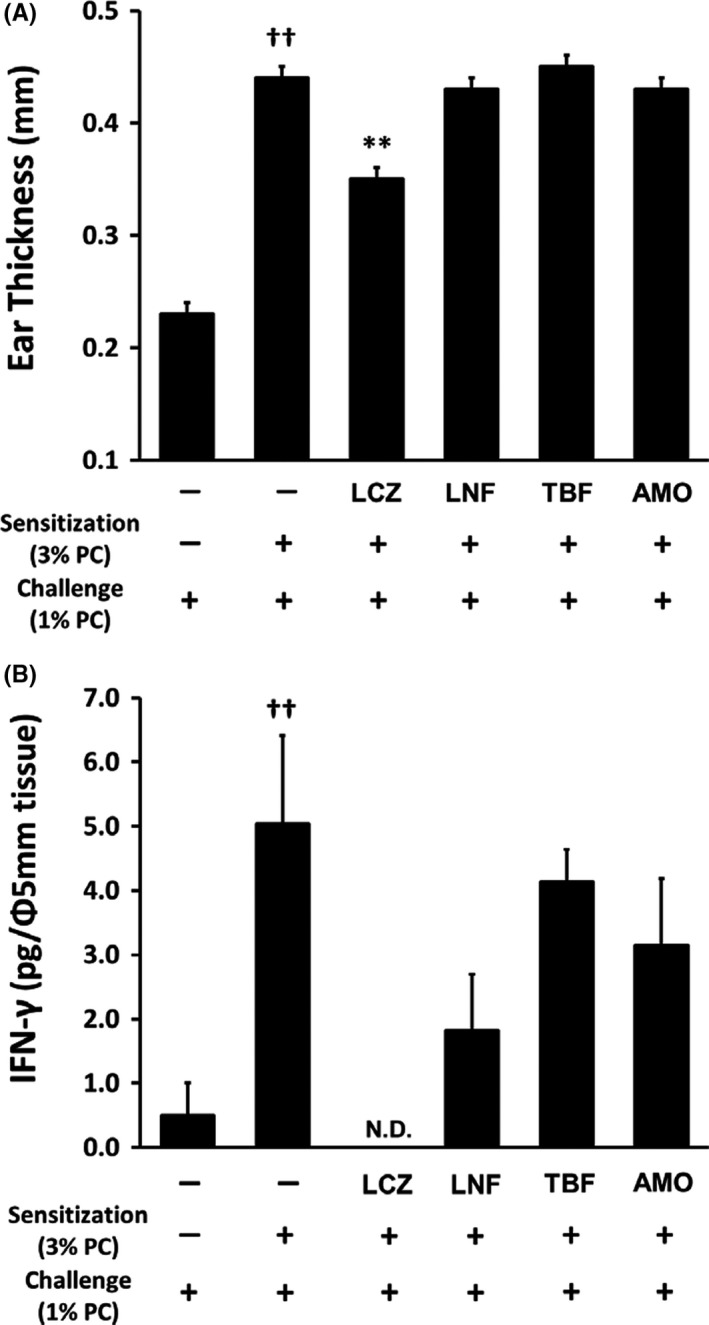
Inhibitory effects of topical antifungal agents on ear swelling of PC‐induced contact dermatitis in mice. Mice were sensitised by topical application of PC (3%, 100 µL) to the shaved abdominal skin. Negative control mice received acetone instead of PC. Six days later, PC (1%, 20 µL) was topically applied to both sides of the right ear. An antifungal agent solution (1%, 20 µL) or vehicle (acetone) was applied immediately after the challenge. (A) The ear thickness was measured using a thickness gauge 24 h after the challenge. (B) Punch biopsies (5‐mm diameter) were obtained after measuring the ear thickness, and the IFN‐γ content in the ear biopsy was measured by enzyme‐linked immunosorbent assay. Each column represents the mean ± standard error of the mean (n = 5). **P < .01 compared with PC alone (Student's *t* test, two‐sided); ^#^ *P* < .05, ^##^ *P* < .01 compared with non‐sensitisation (Student's *t* test, two‐sided). AMO, amorolfine; IFN, interferon; LCZ, lanoconazole; LNF, liranaftate; ND, not detected; PC, 2,4,6‐trinitrophenyl chloride; TBF, terbinafine

We examined the effects of various antifungal agents on PC‐induced contact dermatitis in mice. PC challenge induced a significantly greater increase in ear thickness in sensitised than non‐sensitised mice. Among the various antifungal agents, LCZ significantly inhibited the increase in ear thickness induced by PC (Figure 4A) as previously reported, whereas LNF, TBF and AMO did not suppress the increase in ear thickness induced by PC. Moreover, LCZ markedly inhibited the increase in IFN‐γ content in the ear of the sensitised mice, and the other antifungal agents (LNF, TBF and AMO) showed a tendency to decrease the IFN‐γ content (Figure 4B).

## 4 DISCUSSION

In this study, LCZ significantly inhibited ear swelling associated with TPA‐induced irritant dermatitis in mice at concentrations of more than 1%, and LCZ dose‐dependently and remarkably suppressed the increases in the MPO activity, KC content and MIP‐2 content in TPA‐induced irritant dermatitis. Furthermore, these inhibitory effects of LCZ on TPA‐induced dermatitis were stronger than those of the other antifungal agents. In addition, LCZ exhibited robust inhibition of PC‐induced allergic contact dermatitis in mice compared with the other antifungal agents.

IL‐8 is a representative neutrophil chemotactic factor in humans,^23^ and KC and MIP‐2 are thought to be homologues of human IL‐8 from a functional aspect.^20^, ^21^, ^22^ Actually, KC and MIP‐2 have been shown to be associated with neutrophil migration in various types of inflammatory reactions.^24^, ^25^, ^26^, ^27^, ^28^ Moreover, migration of neutrophils to the skin is significantly reduced by deficiency of CXCR2, which is a receptor of KC and MIP‐2, or by neutralising antibodies against KC and MIP‐2.^29^ These observations suggest that suppression of the production of KC and MIP‐2 by LCZ results in decreased neutrophil migration in TPA‐induced dermatitis. Although the mechanisms by which LCZ inhibits KC and MIP‐2 production remain unknown, the imidazole antifungal agent sertaconazole reportedly inhibits both IL‐8 secretion from human epidermal keratinocytes and ear swelling in TPA‐induced irritant dermatitis in mice by elevating the levels of prostaglandin E_2_ (PGE_2_) via cyclooxygenase‐2 activation, which is mediated by activation of p38 MAPK.^17^ Furthermore, the activation of NF‐κB plays an important role in neutrophil infiltration in mouse skin with TPA‐induced dermatitis, and the expression of KC and MIP‐2 is reduced by inhibiting the activation of NF‐κB.^30^ Ketoconazole, another imidazole antifungal agent, also reportedly inhibits the increase in NF‐κB activity in human epidermal keratinocytes stimulated by tumour necrosis factor‐α, and its inhibitory effect is dependent on PGE_2_ production by keratinocytes.^31^ Therefore, based on these reports of the anti‐inflammatory effects of sertaconazole and ketoconazole, LCZ may also inhibit the production of KC and MIP‐2 by inhibiting the activation of NF‐κB via activation of a p38–cyclooxygenase‐2‐PGE_2_ pathway.

In the present study, we compared the anti‐inflammatory effects of LCZ with those of other antifungal agents, including LNF, TBF and AMO, on TPA‐induced dermatitis in mice. LCZ, LNF, TBF and AMO differ based on the presence of imidazole, thiocarbamic acid, arylamine and morpholine as a basic structure, respectively. As shown in Figure 3, these antifungal agents had different inhibitory effects on ear swelling, MPO activity, and KC and MIP‐2 contents in mouse TPA‐induced dermatitis. We then analysed the relationship between ear swelling and the increase in MPO activity, KC content or MIP‐2 content for each of these antifungal agents to elucidate why the inhibitory effect of LCZ was stronger than that of the other antifungal agents. As expected, we found a significantly positive correlation between the ear thickness and chemokine content or MPO activity, and the correlation coefficient between ear thickness and MPO activity, KC content and MIP‐2 content was 0.74, 0.63 and 0.56, respectively (Figure 3E–G). As previously mentioned, mouse KC and MIP‐2 are the functional analogues of human IL‐8, which is a neutrophil chemotactic factor. LCZ exhibited stronger inhibition of both the increase in KC and MIP‐2 content and MPO activity in the ear skin of mouse TPA‐induced dermatitis than did the other antifungal agents. These findings strongly suggest that LCZ suppresses TPA‐induced irritant dermatitis by inhibiting the production of neutrophil chemotactic factors such as KC or MIP‐2 and the subsequent infiltration of activated neutrophils into the skin.

Furthermore, this study demonstrated the inhibitory effect of LCZ on PC‐induced contact dermatitis in mice as previously reported.^19^ We also found that LCZ reduced the increase in IFN‐γ content in the ear tissue of this dermatitis model. PC‐induced contact dermatitis is thought to be a Th1‐driven immune response, and IFN‐γ produced from T cells is considered to play an important role in the development of dermatitis.^32^ In addition, we previously reported that LCZ inhibited IFN‐γ production by human peripheral blood mononuclear cells in vitro.^19^ These observations suggest that LCZ suppresses PC‐induced contact dermatitis in mice by inhibiting IFN‐γ production from T cells that have infiltrated the skin. Furthermore, whether LCZ can inhibit skin inflammation induced by dermatophytes as well as PC should be examined to further clarify contribution of anti‐inflammatory effect of LCZ to relief of the dermatitis symptoms in patients with tinea pedis, and then in vivo examination using dermatophyte‐induced skin inflammation in mice will need to be done in the next study.

We also compared the effects of LCZ versus other antifungal agents on PC‐induced allergic contact dermatitis in mice. Only LCZ showed inhibitory effects in this dermatitis model, which differs from the results in the TPA‐induced dermatitis model. Although the reason for the discrepancy in the effects of other antifungal agents between the two dermatitis models is not clear, the differences in factors involved in the dermatitis model may affect the results.

In conclusion, the present study demonstrated that LCZ suppressed TPA‐induced irritant dermatitis in mice by inhibiting the production of neutrophil chemotactic factors such as KC and MIP‐2 and neutrophil infiltration at the site of inflammation. Additionally, LCZ inhibited both ear swelling and IFN‐γ production in the ear skin with PC‐induced contact dermatitis in mice, and the anti‐inflammatory effects of LCZ, an imidazole antifungal agent, on these dermatitis models were stronger than those of other types of antifungal agents. These findings suggest that the anti‐inflammatory effects of LCZ may contribute to relief of the dermatitis symptoms in patients with tinea pedis.

## CONFLICT OF INTEREST

All authors are employees of Maruho Co., Ltd.

## AUTHORS’ CONTRIBUTIONS

AN and HU conceived the ideas; HU, YY and KH collected and analysed the data; AN and HU drafted the article; AN, NN and TM reviewed the article and approved its final version.

## References

[1] Weinstein A , Berman B . Topical treatment of common superficial tinea infections. Am Fam Physician. 2002;65:2095‐2102. 12046779

[2] Bell‐Syer SE , Hart R , Crawford F , Torgerson DJ , Tyrrell W , Russell I . Oral treatments for fungal infections of the skin of the foot. Cochrane Database Syst Rev. 2002;CD003584. 12076488 10.1002/14651858.CD003584

[3] Nishimoto K . [An epidemiological survey of dermatomycoses in Japan, 2002]. Nihon Ishinkin Gakkai Zasshi. 2006;47:103‐111. (Japanese). 16699491 10.3314/jjmm.47.103

[4] Nakamura Y , Kano R , Hasegawa A , Watanabe S . Interleukin‐8 and tumor necrosis factor alpha production in human epidermal keratinocytes induced by Trichophyton mentagrophytes. Clin Diagn Lab Immunol. 2002;9:935‐937. 12093702 10.1128/CDLI.9.4.935-937.2002 PMC120037

[5] Tani K , Adachi M , Nakamura Y , et al. The effect of dermatophytes on cytokine production by human keratinocytes. Arch Dermatol Res. 2007;299:381‐387. 17710424 10.1007/s00403-007-0780-7

[6] Koga T , Ishizaki H , Matsumoto T , Hori Y . Cytokine production of peripheral blood mononuclear cells in a dermatophytosis patient in response to stimulation with trichophytin. J Dermatol. 1993;20:441‐443. 8408928 10.1111/j.1346-8138.1993.tb01315.x

[7] Koga T , Shimizu A , Nakayama J . Interferon‐γ production in peripheral lymphocytes of patients with tinea pedis: comparison of patients with and without tinea unguium. Med Mycol. 2001;39:87‐90. 10.1080/mmy.39.1.87.90 11270412

[8] Nakajima H . [The pathophysiology and defense mechanism against superficial and subcutaneous fungal infection]. *Nihon Ishinkin Gakkai* . Zasshi. 2005;46:5‐9.(Japanese). 10.3314/jjmm.46.5 15711529

[9] Marquardt B , Frith D , Stabel S . Signalling from TPA to MAP kinase requires protein kinase C, raf and MEK: reconstitution of signaling pathway in vitro. Oncogene. 1994;9:3213‐3218. 7936644

[10] Sato H , Nakayama Y , Yamashita C , Uno H . Anti‐inflammatory effects of tacalcitol (1,24(R)(OH)2D3, TV‐02) in the skin of TPA‐treated hairless mice. J Dermatol. 2004;31:200‐217. 15187340 10.1111/j.1346-8138.2004.tb00657.x

[11] Sato H , Ogino Y , Takagi H , et al. Pharmacological profiles of high‐concentration (20 microg/g) tacalcitol ointment: effects on cutaneous inflammation, epidermal proliferation, and differentiation in mice. J Dermatol. 2003;30:510‐524. 12928540 10.1111/j.1346-8138.2003.tb00425.x

[12] Plato A , Willment JA , Brown GD . C‐type lectin‐like receptors of the dectin‐1 cluster: ligands and signaling pathways. Int Rev Immunol. 2013;32:134‐156. 23570314 10.3109/08830185.2013.777065 PMC3634610

[13] Baghi N , Shokohi T , Badali H , et al. *In vitro* activity of new azoles luliconazole and lanoconazole compared with ten other antifungal drugs against clinical dermatophyte isolates. Med Mycol. 2016;54:757‐763. 27118804 10.1093/mmy/myw016

[14] Rezaei‐Matehkolaei A , Khodavaisy S , Alshahni MM , et al. *In vitro* antifungal activity of novel triazole efinaconazole and five comparators against dermatophyte isolates. Antimicrob Agents Chemother. 2018;62:e02423‐e2517. 29530856 10.1128/AAC.02423-17 PMC5923135

[15] Rubin AI , Bagheri B , Scher RK . Six novel antimycotics. Am J Clin Dermatol. 2002;3:71‐81. 11893219 10.2165/00128071-200203020-00001

[16] Liebel F , Lyte P , Garay M , Babad J , Southall MD . Anti‐inflammatory and anti‐itch activity of sertaconazole nitrate. Arch Dermatol Res. 2006;298:191‐199. 16868738 10.1007/s00403-006-0679-8

[17] Sur R , Babad JM , Garay M , Liebel FT , Southall MD . Anti‐inflammatory activity of sertaconazole nitrate is mediated via activation of a p38‐COX‐2‐PGE2 pathway. J Invest Dermatol. 2008;128:336‐344. 17637821 10.1038/sj.jid.5700972

[18] Merlos M , Vericat ML , García‐Rafanell J , Forn J . Topical anti‐inflammatory properties of flutrimazole, a new imidazole antifungal agent. Inflamm Res. 1996;45:20‐25. 8821774 10.1007/BF02263500

[19] Uratsuji H , Nakamura A , Yamada Y , et al. Anti‐inflammatory activity of lanoconazole, a topical antifungal agent. Mycoses. 2015;58:197‐202. 25675966 10.1111/myc.12297

[20] Driscoll KE . Macrophage inflammatory proteins: biology and role in pulmonary inflammation. Exp Lung Res. 1994;20:473‐490. 7882902 10.3109/01902149409031733

[21] Bozic CR , Kolakowski LF Jr , Gerard NP , et al. Expression and biologic characterization of the murine chemokine KC. J Immunol. 1995;154:6048‐6057. 7751647

[22] Kobayashi Y . Neutrophil infiltration and chemokines. Crit Rev Immunol. 2006;26:307‐316. 17073556 10.1615/critrevimmunol.v26.i4.20

[23] Mukaida N . Interleukin‐8: an expanding universe beyond neutrophil chemotaxis and activation. Int J Hematol. 2000;72:391‐398. 11197203

[24] Lira SA , Zalamea P , Heinrich JN , et al. Expression of the chemokine N51/KC in the thymus and epidermis of transgenic mice results in marked infiltration of a single class of inflammatory cells. J Exp Med. 1994;180:2039‐2048. 7964481 10.1084/jem.180.6.2039 PMC2191760

[25] Tani M , Fuentes ME , Peterson JW , et al. Neutrophil infiltration, glial reaction, and neurological disease in transgenic mice expressing the chemokine N51/KC in oligodendrocytes. J Clin Invest. 1996;98:529‐539. 8755666 10.1172/JCI118821 PMC507459

[26] Farone A , Huang S , Paulauskis J , Kobzik L . Airway neutrophilia and chemokine mRNA expression in sulfur dioxide‐induced bronchitis. Am J Respir Cell Mol Biol. 1995;12:345‐350. 7873201 10.1165/ajrcmb.12.3.7873201

[27] Greenberger MJ , Strieter RM , Kunkel SL , et al. Neutralization of macrophage inflammatory protein‐2 attenuates neutrophil recruitment and bacterial clearance in murine Klebsiella pneumonia. J Infect Dis. 1996;173:159‐165. 8537653 10.1093/infdis/173.1.159

[28] Seebach J , Bartholdi D , Frei K , et al. Experimental Listeria meningoencephalitis. Macrophage inflammatory protein‐1 alpha and ‐2 are produced intrathecally and mediate chemotactic activity in cerebrospinal fluid of infected mice. J Immunol. 1995;155:4367‐4375. 7594596

[29] Cataisson C , Pearson AJ , Tsien MZ , et al. CXCR2 ligands and G‐CSF mediate PKCalpha‐induced intraepidermal inflammation. J Clin Invest. 2006;116:2757‐2766. 16964312 10.1172/JCI27514 PMC1560349

[30] Cataisson C , Pearson AJ , Torgerson S , Nedospasov SA , Yuspa SH . Protein kinase C alpha‐mediated chemotaxis of neutrophils requires NF‐kappa B activity but is independent of TNF alpha signaling in mouse skin in vivo. J Immunol. 2005;174:1686‐1692. 15661932 10.4049/jimmunol.174.3.1686

[31] Kanda N , Watanabe S . Suppressive effects of antimycotics on tumor necrosis factor‐alpha‐induced CCL27, CCL2, and CCL5 production in human keratinocytes. Biochem Pharmacol. 2006;72:463‐473. 16784723 10.1016/j.bcp.2006.05.001

[32] Kitagaki H , Kimishima M , Teraki Y , et al. Distinct in vivo and in vitro cytokine profiles of draining lymph node cells in acute and chronic phases of contact hypersensitivity: importance of a type 2 cytokine‐rich cutaneous milieu for the development of an early‐type response in the chronic phase. J Immunol. 1999;163:1265‐1273. 10415023

